# Noninvasive Prenatal Diagnosis of Fetal Trisomy 18 and Trisomy 13 by Maternal Plasma DNA Sequencing

**DOI:** 10.1371/journal.pone.0021791

**Published:** 2011-07-06

**Authors:** Eric Z. Chen, Rossa W. K. Chiu, Hao Sun, Ranjit Akolekar, K. C. Allen Chan, Tak Y. Leung, Peiyong Jiang, Yama W. L. Zheng, Fiona M. F. Lun, Lisa Y. S. Chan, Yongjie Jin, Attie T. J. I. Go, Elizabeth T. Lau, William W. K. To, Wing C. Leung, Rebecca Y. K. Tang, Sidney K. C. Au-Yeung, Helena Lam, Yu Y. Kung, Xiuqing Zhang, John M. G. van Vugt, Ryoko Minekawa, Mary H. Y. Tang, Jun Wang, Cees B. M. Oudejans, Tze K. Lau, Kypros H. Nicolaides, Y. M. Dennis Lo

**Affiliations:** 1 Centre for Research into Circulating Fetal Nucleic Acids, Li Ka Shing Institute of Health Sciences, The Chinese University of Hong Kong, Hong Kong SAR, China; 2 Department of Chemical Pathology, The Chinese University of Hong Kong, Hong Kong SAR, China; 3 Department of Obstetrics and Gynaecology, The Chinese University of Hong Kong, Hong Kong SAR, China; 4 Harris Birthright Research Centre for Fetal Medicine, King's College Hospital, London, United Kingdom; 5 VU University Medical Center, Amsterdam, The Netherlands; 6 Tsan Yuk Hospital, Department of Obstetrics and Gynaecology, University of Hong Kong, Hong Kong SAR, China; 7 United Christian Hospital, Hong Kong SAR, China; 8 Kwong Wah Hospital, Hong Kong SAR, China; 9 Pamela Youde Nethersole Eastern Hospital, Hong Kong SAR, China; 10 Tuen Mun Hospital, Hong Kong SAR, China; 11 Princess Margaret Hospital, Hospital Authority, Hong Kong SAR, China; 12 YY Kung Medical Centre, Hong Kong SAR, China; 13 Joint Chinese University of Hong Kong-Beijing Genomics Institute Genome Research Centre, Li Ka Shing Institute of Health Sciences, Hong Kong SAR, China; 14 Beijing Genomics Institute at Shenzhen, Shenzhen, China; Innsbruck Medical University, Austria

## Abstract

Massively parallel sequencing of DNA molecules in the plasma of pregnant women has been shown to allow accurate and noninvasive prenatal detection of fetal trisomy 21. However, whether the sequencing approach is as accurate for the noninvasive prenatal diagnosis of trisomy 13 and 18 is unclear due to the lack of data from a large sample set. We studied 392 pregnancies, among which 25 involved a trisomy 13 fetus and 37 involved a trisomy 18 fetus, by massively parallel sequencing. By using our previously reported standard z-score approach, we demonstrated that this approach could identify 36.0% and 73.0% of trisomy 13 and 18 at specificities of 92.4% and 97.2%, respectively. We aimed to improve the detection of trisomy 13 and 18 by using a non-repeat-masked reference human genome instead of a repeat-masked one to increase the number of aligned sequence reads for each sample. We then applied a bioinformatics approach to correct GC content bias in the sequencing data. With these measures, we detected all (25 out of 25) trisomy 13 fetuses at a specificity of 98.9% (261 out of 264 non-trisomy 13 cases), and 91.9% (34 out of 37) of the trisomy 18 fetuses at 98.0% specificity (247 out of 252 non-trisomy 18 cases). These data indicate that with appropriate bioinformatics analysis, noninvasive prenatal diagnosis of trisomy 13 and trisomy 18 by maternal plasma DNA sequencing is achievable.

## Introduction

Trisomy 13 (Patau syndrome) and trisomy 18 (Edwards syndrome) are the most clinically important autosomal trisomies besides trisomy 21. Trisomy 13 occurs in about 1 out of every 10,000 newborns and the incidence of trisomy 18 is estimated to be 1 in 6,000 live births [1].

Detection of such fetal chromosomal aberrations is an important indication for prenatal diagnosis. Conventional prenatal diagnostic methods, such as sampling of fetal genetic materials by amniocentesis or chorionic villus sampling, are invasive, and carry potential risks to the fetus [2]. Besides these invasive approaches, noninvasive screening approaches by ultrasound scanning and maternal serum markers are useful for identifying high-risk cases, but have limited sensitivity and specificity. For example, the detection rate of first-trimester combined screening is 77%–86% and the false positive rate is 3.2%–5.6% [3]. These approaches measure epiphenomena associated with the trisomies, rather than directly detecting the core abnormality involving chromosomal dosage [3], [4].

Cell-free fetal DNA has been shown to be present in the plasma of pregnant women [5], and has opened up new possibilities for noninvasive prenatal diagnosis [6]. Tests based on circulating fetal DNA have been used clinically for the prenatal management of sex-linked disorders and rhesus D incompatibility [7]–[9]. Recently, strategies have also been developed for the noninvasive diagnosis of fetal aneuploidy by fetal nucleic acid analysis in maternal plasma, including the detection of fetal DNA methylation signatures and mRNA markers in maternal plasma [10]–[15].

In addition, when a woman is pregnant with a trisomic fetus, there should be an increased proportion, i.e. over representation, of fetal-derived DNA molecules from the extra chromosome in her plasma when compared to a pregnancy with a euploid fetus. With the availability of massively parallel sequencing (MPS) technologies which sequence DNA molecules in a high throughput manner, the genomic identities and quantities of millions of DNA molecules in biological samples could be determined. It has been shown that sequencing of maternal plasma DNA can be applied to the noninvasive detection of fetal trisomy 21 [16]–[19]. Essentially, maternal plasma DNA molecules are sequenced, and the chromosomal origin of each sequenced molecule is identified by comparing to a reference human genome sequence. The number of molecules derived from chromosome 21 (chr21) as a proportion of all sequenced molecules has been shown to be elevated in trisomy 21 pregnancies when compared with euploid ones. Our group has demonstrated that detection of fetal trisomy 21 could be achieved using two different MPS platforms, namely the sequencing-by-synthesis (SBS) platform [16] and the sequencing-by-ligation (SBL) platform [17]. Two recent large scale studies on trisomy 21 detection based on SBS platform both showed a 100% sensitivity and 97.9% to 99.7% specificity [19], [20].

Theoretically, if there were no analytical or biological bias of MPS, it would be expected that the molecules from the whole genome could be sequenced uniformly by the procedure. However, it has been reported that molecules from different regions of a genome may not be uniformly sequenced by MPS. The guanine and cytosine (GC) content of the sequenced nucleic acids has been reported to contribute to the non-uniformity [21], [22]. Fan et al. and our group found that the patterns of non-uniform representation of each chromosome were different when maternal plasma DNA was sequenced with the use of different MPS platforms [17], [18]. These data suggest that the non-uniform representation of each chromosome is more likely to be due to sequencing or alignment bias than biological reasons [17], [21]. Furthermore, in our previous study, we have highlighted that chr13 and chr18 have relatively lower average GC content than chr21 which has a modest average GC content [16]. In addition, we have previously shown that the measurement of the genomic representations of chr13 and chr18 is less precise than that for chr21 based on both the SBS and SBL platforms [16], [17]. The variance of measuring the genomic representations of chr13 and chr18, as indicated by the coefficient of variation (CV), are 5.8 times and 2.4 times, respectively, greater than that of chr21 on the SBS platform [16]. Similar results were also observed on the SBL platform on which the variance of measuring the genomic representations of chr13 and chr18 are 5.7 times and 1.8 times, respectively, greater than that of chr21 [17]. These observations have led us to hypothesize that noninvasive prenatal diagnosis of trisomy 13 and trisomy 18 by MPS would likely be less accurate than for trisomy 21 [17]. As the variance in the GC content of the sequenced molecules is a contributor to the issue, quantitative correction to minimize such an effect on the measurement of the genomic representations of chr13 and chr18 might allow accurate diagnosis of trisomy 13 and trisomy 18.

To this end, here we assessed the prenatal diagnostic performance by MPS of maternal plasma DNA on a cohort of 392 pregnancies of whom 25 involved trisomy 13 fetuses and 37 involved trisomy 18 fetuses.

## Methods

### Ethics Statement

Approvals were obtained from the institutional review boards of each recruitment site: Joint Chinese University of Hong Kong-New Territories East Cluster Clinical Research Ethics Committee, Joint Institutional Review Board of the University of Hong Kong-Hospital Authority Hong Kong West Cluster, Clinical and Research Ethics Committees of the Hospital Authority in the Kowloon Central/Kowloon East, Kowloon West, Hong Kong East, New Territories West Clusters, King's College Hospital Ethics Committee and Ethics Committee of the VU University Medical Center. All participants gave informed written consent.

### Study design, setting and population

252 participants were recruited prospectively from Hong Kong, the Netherlands and UK. 140 archived maternal plasma samples from pregnancies with and without trisomy 13, 18 or 21, matched for gestational ages were also retrieved from the participating sites in the Netherlands and UK. Samples that passed the inclusion criteria as described in our previous investigation on the prenatal diagnosis of trisomy 21 [20] were used in this study. Only singleton pregnancies with clinical indications for amniocentesis or CVS were recruited and samples with full karyotypes were included.

**Figure 1:**
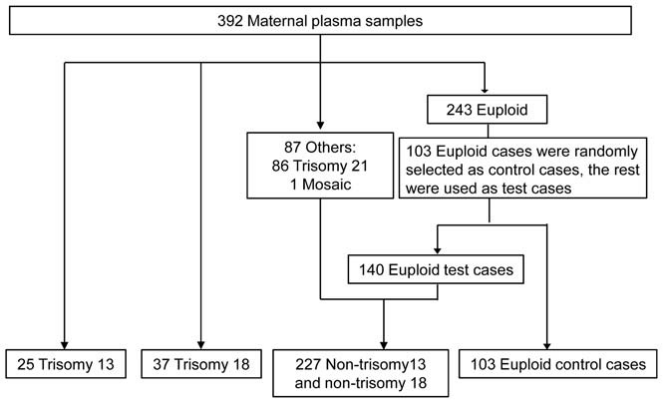
Recruitment of samples.

A total of 392 cases were analyzed, including 25 cases each involving a trisomy 13 fetus, 37 cases each involving a trisomy 18 fetus and 86 cases each involving a trisomy 21 fetus (Figure 1). 314 of the cases were those analyzed using 2-plex sequencing (see below) in a recent study on the noninvasive diagnosis of trisomy 21 [20]. Another 30 cases were from those analyzed using 8-plex sequencing by Chiu et al [20], but were re-sequenced in this study using 2-plex sequencing. The remaining 48 cases were newly recruited and sequenced in this study. 91 cases in the previously recruited cases and 12 cases in the newly recruited cases, which were from women pregnant with a euploid fetus, were used as normal controls.

### Maternal plasma DNA sequencing

Plasma samples from participating pregnant women were processed as previously described [16], [20]. Briefly, five to ten milliliters of peripheral venous blood was collected from each participating pregnant woman in ethylenediaminetetraacetic acid (EDTA)-containing tubes. The blood samples were first centrifuged at 1,600 *g* for 10 min at 4°C so as to separate the plasma from the peripheral blood cells. The plasma portion was carefully transferred to plain polypropylene tubes and then subjected to centrifugation at 16,000 *g* for 10 min at 4°C to pellet the remaining cells [23]. Plasma DNA from 2 to 4.8 milliliters of maternal plasma was extracted by using the QIAamp DSP DNA blood mini kit (Qiagen, Hilden, Germany) following the blood and body fluid protocol.

The DNA library for the extracted plasma DNA was constructed using the Genomic DNA Library Preparation Kit and Multiplexing Sample Preparation Oligonucleotide kit (Illumina) mostly according to manufacturer's instructions. Since plasma DNA molecules are short fragments in nature [24]; hence, the steps of fragmentation and size selection by gel electrophoresis were omitted. Multiplexing was achieved by introducing a 6-bp index barcode to the adapter-ligated DNA of each plasma sample through a triple-primer PCR amplification.

Indexed DNA libraries were sequenced using the 2-plex strategy [20]. 2-plex multiplexing allows two plasma samples to be sequenced in each lane by using different short index sequences (6 bp) for different samples. The index sequences were randomly assigned to the sequencing samples and had at least three-nucleotide differences between each other. A total of 16 specimens could be sequenced in each run. DNA clusters were generated using an Illumina cluster station according to manufacturer's instructions. We sequenced 36 basepairs (bp) from one end of each plasma DNA molecule. An additional 7 cycles of sequencing were performed to decode the index sequence on each sequenced plasma DNA molecule. The sequencing was performed on the Genome Analyzer IIx (Illumina) using the Sequencing Kit V3 (Illumina).

Samples from women pregnant with male euploid fetuses were used as controls for trisomy detection. Four control samples were sequenced in each sequencing run. Thus, in each 2-plex sequencing run, the remaining 12 samples (either trisomy or euploid) were test samples. Samples from women pregnant with trisomic fetuses were identified by comparing the test samples with control samples within each sequencing run by a specific statistical approach. Two statistical approaches have been developed for the diagnosis of trisomy 13 and trisomy 18. One is our previously reported approach (named the standard z-score approach) which was originally designed for trisomy 21 diagnosis [16]. The other was based on z-score calculation but with an additional GC correction step (named the z-score approach with GC correction). Diagnostic performance of these two approaches was assessed by comparing with the results of full karyotyping of the amniotic fluid or chorionic villus samples.

### Sequencing data analysis

The 36-bp read from one end of each sequenced plasma DNA molecule was first mapped to the repeat-masked reference human genome (NCBI Build 36 version 48) using the Short Oligonucleotide Alignment Program 2 (SOAP2) [25] with no mismatches allowed. Then, in order to increase the number of aligned reads for each sample, we also mapped the reads to the non-repeat-masked reference human genome (Hg18 NCBI.36). We only used the “unique perfectly aligned reads”, defined as reads that could be mapped to only one location without any mismatch in the reference human genome, for further analysis. Due to the 2-plex sequencing strategy, the reads in each lane were then sorted back to the corresponding samples according to the index DNA sequence information with one mismatch allowed in the index DNA sequences.

### Detection of trisomy 13 and trisomy 18

We counted the number of aligned reads for each chromosome. The genomic representations of chr13 and chr18 are defined as the percentages of the aligned reads originated from chr13 and chr18, respectively, among the total reads sequenced from a sample. A maternal plasma sample from a pregnancy with a trisomy 13/18 fetus is expected to have a higher genomic representation of chr13/18 compared with a set of reference samples (samples from women pregnant with euploid fetuses). In order to quantitatively measure this overrepresentation for each tested sample, for the standard z-score approach, the z-score for the chromosome of interest (chr13/18) was calculated by the following equation:
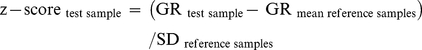
(1)


where GR _test sample_ is the genomic representation of the chromosome of interest in the test sample; GR _mean reference samples_ is the mean of the genomic representations of the chromosome of interest for all reference samples; and SD _reference samples_ is the standard deviation of the genomic representations of the chromosome of interest for the reference samples.

For the z-score approach with GC correction, instead of using read counts to directly calculate the genomic representations of the chromosomes of interest, a GC-corrected read count was used to calculate the genomic representations with an aim to eliminate the effect of the GC bias on the sequenced read counts. For each sample, all chromosomes were first bioinformatically divided into segments of the same size, called bins. In preliminary analyses, we tested bin sizes of 50 kb, 100 kb, 500 kb and 1000 kb. We found that the CVs for measuring chr13 and chr18 in maternal plasma did not vary significantly using these bin sizes. Thus, we chose 50 kb as the bin size for all subsequent analyses. The number of sequenced reads and GC content in each bin (rounded to 0.1%) were determined. We filtered bins without any reads and bins with ‘N’ in the sequences. Then, locally weighted scatterplot smoothing (LOESS) regression was applied to fit the number of sequenced reads in each bin against the GC content of the corresponding bin in order to calculate the LOESS fit predicted value (P) [22]. Using the regression function and the GC content of each bin, we obtained the LOESS fit predicted value for each bin. The GC corrected read counts (RC_GC_) of each bin were calculated from the raw read counts (RC_raw_) with the correction factor (F). The latter was derived from the median counts of all the bins (M) and the LOESS fit predicted value by the following equations:
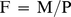
(2)

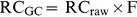
(3)After GC correction, the z-score statistic was calculated by using the genomic representations derived from the GC-corrected read counts of chr13/18 with equation 1.

For both approaches, a z-score value greater than 3, representing a genomic representation greater than that of the 99.9^th^ percentile of the reference set for a one-tailed distribution, was used as the cut-off to determine if overrepresentation of chr13/18 plasma DNA molecules and hence fetal trisomy 13/18 was present.

### Statistical analysis

We reported the diagnostic sensitivity and specificity values of the standard z-score approach and the z-score approach with GC correction, using the z-score value of 3 as the cutoff. LOESS normalization procedure was performed by R (http://www.r-project.org/) with default parameters. Spearman correlation coefficient analysis and z-score calculation were also performed by R.

## Results

### Study participants

392 pregnancies were studied (Figure 1). Amongst these, 243 had euploid fetuses. 103 of these samples were used as reference control samples for z-score calculation. Amongst these, 90 and 13 euploid cases were used as reference controls in one and two flow cells, respectively. To study the detection of the two trisomies, 25 trisomy 13 cases were compared with 264 non-trisomy 13 cases (consisting of 140 euploid cases not used as reference controls, 86 trisomy 21 cases, 1 sex chromosome mosaic case and 37 trisomy 18 cases). Similarly, 37 trisomy 18 cases were compared with 252 non-trisomy 18 cases (consisting of 140 euploid cases not used as reference controls, 86 trisomy 21 cases, 1 sex chromosome mosaic case and 25 trisomy 13 cases).

### Diagnostic performance of the standard z-score approach

**Figure 2:**
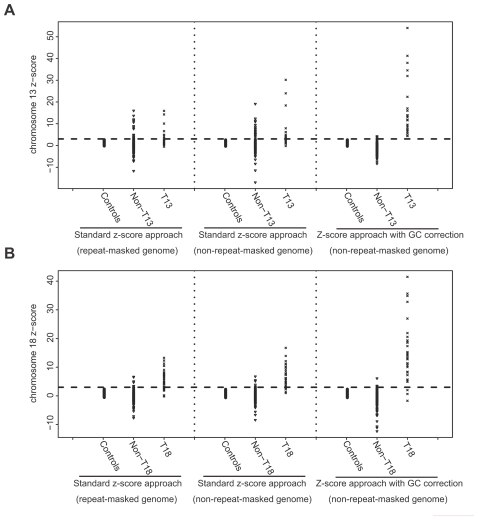
Z-scores of chromosome 13 and 18 determined by the standard z-score approach and the z-score approach with GC correction. Z-scores of (**A**) chromosome 13 and (**B**) chromosome 18 determined by the standard z-score approach with alignment against the repeat-masked or non-repeat-masked reference genomes and the z-score approach with GC correction are shown. Horizontal dashed lines indicate the z-score cut off value of 3. T13, trisomy 13. T18, trisomy 18.

We first tested our previously reported standard z-score approach for trisomy 13 and trisomy 18 detection. We first aligned all the reads to the repeat-masked reference human genome as we did in our previous study [16], [20]. After alignment, we obtained a mean of 2.4 million (SD 508,842) unique perfectly aligned reads per maternal plasma sample. The average per base coverage per genome is 0.028 (SD 0.006). The diagnostic performance of the standard z-score approach for detecting fetal trisomy 13 and trisomy 18 is shown in Tables 1, 2 and 3. The z-scores of chr13 and chr18 are shown in Figures 2A and 2B. Using a chr13/18 z-score value of 3 as the cutoff, only 9 of the 25 trisomy 13 cases were detected. In addition, 244 of the 264 non-trisomy 13 cases were classified correctly. In other words, the sensitivity and specificity for detecting trisomy 13 were 36.0% and 92.4%, respectively. For the detection of the trisomy 18 cases, 27 of 37 trisomy 18 cases and 245 of 252 non-trisomy 18 cases were classified correctly, giving a sensitivity and specificity of 73.0% and 97.2%, respectively.

**Table 1:** Actual and predicted outcome of maternal plasma DNA sequencing for fetal trisomy 13 detection.

		Standard z-score approach		Standard z-score approach		Z-score approach with GC correction	
		(repeat-masked genome)		(non-repeat-masked genome)		(non-repeat-masked genome)	
		Predicted		Predicted		Predicted	
		T13	Non-T13	T13	Non-T13	T13	Non-T13
Actual	T13	9	16	11	14	25	0
	Non-T13	20	244	17	247	3	261

T13: trisomy 13. chr13 z-score >3 was used as the diagnostic cut-off.

**Table 2:** Actual and predicted outcome of maternal plasma DNA sequencing for fetal trisomy 18 detection.

		Standard z-score approach		Standard z-score approach		Z-score approach with GC correction	
		(repeat-masked genome)		(non-repeat-masked genome)		(non-repeat-masked genome)	
		Predicted		Predicted		Predicted	
		T18	Non-T18	T18	Non-T18	T18	Non-T18
Actual	T18	27	10	31	6	34	3
	Non-T18	7	245	5	247	5	247

T18, trisomy 18. chr18 z-score >3 was used as the diagnostic cut-off.

**Table 3:** Diagnostic performance of maternal plasma DNA sequencing for fetal trisomy 13 and 18 detection.

	Standard z-score approach		Standard z-score approach		Z-score approach with GC correction	
	(repeat-masked genome)		(non-repeat-masked genome)		(non-repeat-masked genome)	
	T13	T18	T13	T18	T13	T18
Sensitivity:	36.0%	73.0%	44.0%	83.8%	100.0%	91.9%
Specificity:	92.4%	97.2%	93.6%	98.0%	98.9%	98.0%
PPV:	31.0%	79.4%	39.3%	86.1%	89.3%	87.2%
NPV:	93.8%	96.1%	94.6%	97.6%	100.0%	98.8%

PPV: positive predictive value, NPV: negative predictive value, T13: trisomy 13, T18, trisomy 18. chr13 or 18 z-score >3 was used as the diagnostic cut-off.

The diagnostic performances for trisomy 13 and 18 detection were worse than that for trisomy 21 which was 100% sensitive and 97.9% specific [20]. We therefore investigated a number of bioinformatics approaches to try to improve the classification accuracies for trisomy 13 and 18. As the statistical power of the molecular counting approach used in the current study increased with the number of counted molecules [26], we attempted to increase the number of aligned reads by using the non-repeat-masked reference human genome for alignment. Consequently, we obtained a mean of 4.6 million (SD 964,094) unique perfectly aligned reads per maternal plasma sample. The average per base coverage per genome is 0.053 (SD 0.011). The diagnostic performances of the standard z-score approach for detecting fetal trisomy 13 and trisomy 18 using these increased aligned read counts are shown in Tables 1, 2 and 3. The z-scores of chr13 and chr18 are shown in Figures 2A and 2B. For trisomy 13 detection, 11 of 25 trisomy 13 cases and 247 of 264 non-trisomy 13 cases were identified correctly, corresponding to improved sensitivity and specificity of 44.0% and 93.6%, respectively. For trisomy 18, 31 of 37 trisomy 18 cases and 247 of 252 non-trisomy 18 cases were identified correctly, corresponding to sensitivity and specificity of 83.8% and 98.0%, respectively.

### Diagnostic performance of the z-score approach with GC correction

Although the detection accuracies of trisomy 13 and trisomy 18 were improved by using the non-repeat masked reference genome for alignment, these figures still had considerable room for improvement. We thus attempted to further improve the classification accuracies for trisomy 13 and trisomy 18 by applying a GC correction algorithm. For the z-score approach with GC correction, when using the same diagnostic z-score value of 3, all the trisomy 13 cases (25 out of 25) were successfully identified, while 261 out of 264 non-trisomy 13 cases were correctly classified (Figure 2A and Table 1). The sensitivity and specificity of this approach were thus 100% and 98.9%, respectively (Table 3). For trisomy 18, 34 out of 37 trisomy 18 cases and 247 out of 252 non-trisomy 18 cases were correctly identified (Figure 2B and Table 2), corresponding to sensitivity and specificity of 91.9% and 98.0%, respectively (Table 3).

### GC correction improved the precision for measuring genomic representations of chr13 and chr18 in plasma

The improved diagnostic performance of the z-score approach with GC correction compared with the standard z-score approach is likely to be due to amelioration of the sequencing GC bias and the more precise measurement of the genomic representations of chr13 and chr18 (the percentage of read counts from chr13 and chr18 over the total read count) in maternal plasma.

As there is a strong positive correlation between read counts and the average GC content of all bins (average Spearman correlation coefficient for all the samples  = 0.56, SD  = 0.13, all p value <1.0×10^−10^ for all samples except one), the difference in GC content between different chromosomes would likely lead to quantitative biases in the genomic representations of fragments from different chromosomes and hence affect the precision of their measurement in plasma [17].

**Figure 3:**
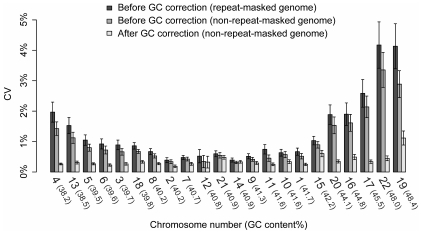
Precision before and after GC correction for the autosomes. Coefficient of variation (CV) for each chromosome was calculated based on the control euploid cases before GC correction with alignment against the repeat-masked or non-repeat-masked reference genomes and after GC correction with alignment against the non-repeat-masked reference genome. Chromosomes are ordered from left to right in increasing GC contents. GC content of each chromosome is shown in the brackets. 95% confidence interval was shown in error bars.

Thus, we calculated the CV for measuring the genomic representation of each autosome among the euploid control samples within each sequencing run. As shown in Figure 3, the autosomes with high or low GC content tend to exhibit a higher variance than autosomes with average GC content. The average CVs for measuring the genomic representations of chromosomes 13 and 18 based on our previous analysis strategy (repeat-masked genome without GC correction) were 1.5% and 0.86%, respectively. When the non-repeat-masked reference genome was used, the CVs for chromosomes 13 and 18 were 1.1% and 0.66%, respectively. After further applying GC correction, the CVs for measuring the genomic representations of chromosomes 13 and 18 decreased to 0.31% and 0.33%, representing overall reductions of 79.7% and 61.2%, respectively. These results indicate that our analysis strategy has markedly improved the precision of quantification of chromosome 13 and chromosome 18, and would therefore result in greater differentiation in the z-score values between the respective trisomy and non-trisomy cases.

## Discussion

This study reports a large series of trisomy 13 and trisomy 18 cases assessed for noninvasive prenatal diagnosis by MPS. We showed that the use of the non-repeat-masked genome as reference and the GC correction approach improved the accuracy of trisomy 13 and trisomy 18 diagnosis based on maternal plasma DNA sequencing.

In essence, accurate diagnosis of trisomy 13 and trisomy 18 could be achieved by having more aligned reads in general. Besides using the non-repeat-masked genome as the alignment reference, another way to increase the number of aligned reads is to perform deeper sequencing. However, the sequencing cost would also increase along with the more sequencing reads. Therefore, increasing the number of aligned reads in a bioinformatics way, as described in this study, is relatively more cost-effective.

The sequencing read length is a potential factor which could alter the proportion of the aligned reads. In this study, we used 36-bp sequenced reads for our application since they were long enough to be aligned to the reference genome and the sequencing time required was short enough to meet the required turnaround time for a diagnostic test. It has been shown that reads with 35-bp length can be uniquely aligned to the human genome at an alignment rate of 85.4% [27]. Although longer reads may contribute to slightly higher alignment [27], the tradeoff is a longer sequencing time. On the other hand, paired-end reads may improve the alignment accuracy but also requires longer sequencing time. Other factors which might be usefully investigated in future work would include improvements in plasma DNA isolation method and technologies for enriching fetal DNA based on size selection [24], [28].

We observed that the improvement for trisomy 13 was more marked than that for trisomy 18, probably because of the more significant deviation in the GC content for chr13 (around 38.5%) when compared with a chromosome with average GC content (e.g. chr21, with GC content of 40.9%). In comparison, chr18 has a GC content (39.8%) much closer to that of chr21.

Besides the GC correction method described in this study, we have also started exploring another approach to reduce the GC bias by assembling an ‘artificial reference genome’ with genomic regions selected for specific GC content characteristics. Future work would shed light with regard to the relative merits of these approaches.

Fan et al. also reported a study on the diagnosis of trisomy 13 and trisomy 18 by maternal plasma DNA sequencing [29]. However, compared with our study involving 25 samples from women pregnant with trisomy 13 fetuses and 37 samples from pregnant women with trisomy 18 fetuses, their test sample size is small which included only 2 trisomy 18 cases and 1 trisomy 13 case. With such a small sample size, it was difficult to conclusively ascertain the diagnostic accuracies of their procedures. Furthermore, the samples in their study were collected soon after amniocentesis or chorionic villus sampling, which might result in an increase of fetal DNA concentration in maternal circulation [30]. Conversely, our much larger study was based on samples collected before amniocentesis or chorionic villus sampling.

In conclusion, maternal plasma DNA sequencing with statistical correction can accurately detect fetal trisomy 13 and trisomy 18 with high detection rate and low false positive rate. Currently, the laboratory cost of our approach including the cost for sequencing reagents and labor is around USD 1000 per case. However, the sequencing cost is expected to rapidly decrease in the future. Furthermore, we have also recently shown that maternal plasma DNA sequencing can also allow one to construct a genome-wide genetic and mutational map of the fetus. Thus, we believe that plasma DNA sequencing would play an increasingly important part of future obstetrics care.

## References

[1] Driscoll DA, Gross S (2009). Clinical practice. Prenatal screening for aneuploidy.. N Engl J Med.

[2] Tabor A, Philip J, Madsen M, Bang J, Obel EB (1986). Randomised controlled trial of genetic amniocentesis in 4606 low-risk women.. Lancet.

[3] Malone FD, Canick JA, Ball RH, Nyberg DA, Comstock CH (2005). First-trimester or second-trimester screening, or both, for Down's syndrome.. N Engl J Med.

[4] Wapner R, Thom E, Simpson JL, Pergament E, Silver R (2003). First-trimester screening for trisomies 21 and 18.. N Engl J Med.

[5] Lo YMD, Corbetta N, Chamberlain PF, Rai V, Sargent IL (1997). Presence of fetal DNA in maternal plasma and serum.. Lancet.

[6] Lo YMD, Chiu RWK (2007). Prenatal diagnosis: progress through plasma nucleic acids.. Nat Rev Genet.

[7] Finning K, Martin P, Summers J, Massey E, Poole G (2008). Effect of high throughput RHD typing of fetal DNA in maternal plasma on use of anti-RhD immunoglobulin in RhD negative pregnant women: prospective feasibility study.. BMJ.

[8] Wright CF, Chitty LS (2009). Cell-free fetal DNA and RNA in maternal blood: implications for safer antenatal testing.. BMJ.

[9] Lo YMD, Hjelm NM, Fidler C, Sargent IL, Murphy MF (1998). Prenatal diagnosis of fetal RhD status by molecular analysis of maternal plasma.. N Engl J Med.

[10] Tong YK, Ding C, Chiu RWK, Gerovassili A, Chim SSC (2006). Noninvasive prenatal detection of fetal trisomy 18 by epigenetic allelic ratio analysis in maternal plasma: Theoretical and empirical considerations.. Clin Chem.

[11] Tong YK, Jin S, Chiu RWR, Ding C, Chan KCA (2009). Noninvasive prenatal detection of trisomy 21 by an epigenetic-genetic chromosome-dosage approach.. Clin Chem.

[12] Tsui NBY, Wong BCK, Leung TY, Lau TK, Chiu RWK (2009). Non-invasive prenatal detection of fetal trisomy 18 by RNA-SNP allelic ratio analysis using maternal plasma SERPINB2 mRNA: a feasibility study.. Prenat Diagn.

[13] Lo YMD, Lun FMF, Chan KCA, Tsui NBY, Chong KC (2007). Digital PCR for the molecular detection of fetal chromosomal aneuploidy.. Proc Natl Acad Sci U S A.

[14] Lo YMD, Tsui NBY, Chiu RWK, Lau TK, Leung TN (2007). Plasma placental RNA allelic ratio permits noninvasive prenatal chromosomal aneuploidy detection.. Nat Med.

[15] Papageorgiou EA, Fiegler H, Rakyan V, Beck S, Hulten M (2009). Sites of differential DNA methylation between placenta and peripheral blood: molecular markers for noninvasive prenatal diagnosis of aneuploidies.. Am J Pathol.

[16] Chiu RWK, Chan KCA, Gao Y, Lau VYM, Zheng W (2008). Noninvasive prenatal diagnosis of fetal chromosomal aneuploidy by massively parallel genomic sequencing of DNA in maternal plasma.. Proc Natl Acad Sci U S A.

[17] Chiu RWK, Sun H, Akolekar R, Clouser C, Lee C (2010). Maternal plasma DNA analysis with massively parallel sequencing by ligation for noninvasive prenatal diagnosis of trisomy 21.. Clin Chem.

[18] Fan HC, Blumenfeld YJ, Chitkara U, Hudgins L, Quake SR (2008). Noninvasive diagnosis of fetal aneuploidy by shotgun sequencing DNA from maternal blood.. Proc Natl Acad Sci U S A.

[19] Ehrich M, Deciu C, Zwiefelhofer T, Tynan JA, Cagasan L (2011). Noninvasive detection of fetal trisomy 21 by sequencing of DNA in maternal blood: a study in a clinical setting.. Am J Obstet Gynecol.

[20] Chiu RWK, Akolekar R, Zheng YW, Leung TY, Sun H (2011). Non-invasive prenatal assessment of trisomy 21 by multiplexed maternal plasma DNA sequencing: large scale validity study.. BMJ.

[21] Dohm JC, Lottaz C, Borodina T, Himmelbauer H (2008). Substantial biases in ultra-short read data sets from high-throughput DNA sequencing.. Nucleic Acids Res.

[22] Alkan C, Kidd JM, Marques-Bonet T, Aksay G, Antonacci F (2009). Personalized copy number and segmental duplication maps using next-generation sequencing.. Nat Genet.

[23] Chiu RWK, Poon LL, Lau TK, Leung TN, Wong EM (2001). Effects of blood-processing protocols on fetal and total DNA quantification in maternal plasma.. Clin Chem.

[24] Chan KCA, Zhang J, Hui AB, Wong N, Lau TK (2004). Size distributions of maternal and fetal DNA in maternal plasma.. Clin Chem.

[25] Li R, Yu C, Li Y, Lam TW, Yiu SM (2009). SOAP2: an improved ultrafast tool for short read alignment.. Bioinformatics.

[26] Chiu RWK, Cantor CR, Lo YMD (2009). Non-invasive prenatal diagnosis by single molecule counting technologies.. Trends Genet.

[27] Li R, Li Y, Fang X, Yang H, Wang J (2009). SNP detection for massively parallel whole-genome resequencing.. Genome Res.

[28] Lo YMD, Chan KCA, Sun H, Chen EZ, Jiang P (2011). Maternal plasma DNA sequencing reveals the genome-wide genetic and mutational profile of the fetus.. Sci Transl Med.

[29] Fan HC, Quake SR (2010). Sensitivity of noninvasive prenatal detection of fetal aneuploidy from maternal plasma using shotgun sequencing is limited only by counting statistics.. PLoS One.

[30] Samura O, Miharu N, Hyodo M, Honda H, Ohashi Y (2003). Cell-free fetal DNA in maternal circulation after amniocentesis.. Clin Chem.

